# The importance of different forest management systems for people’s dietary quality in Tanzania

**DOI:** 10.1007/s10980-024-01961-6

**Published:** 2024-09-11

**Authors:** R. S. Olesen, F. Reiner, B. den Braber, C. Hall, C. J. Kilawe, J. Kinabo, J. Msuya, L. V. Rasmussen

**Affiliations:** 1 Department of Geosciences and Natural Resource Management, University of Copenhagen, Øster Voldgade 10, 1350 Copenhagen, Denmark; 2 Biological and Environmental Sciences, University of Stirling, Stirling, FK9 4LA UK; 3 Department of Ecosystems and Conservation, Sokoine University of Agriculture, Morogoro, Tanzania; 4 Department of Human Nutrition and Consumer Sciences, Sokoine University of Agriculture, Morogoro, Tanzania

**Keywords:** Food and nutrition security, Nutrient adequacy, Dietary quality, Forest management, Tree cover, Multi-functional landscape

## Abstract

**Context:**

A large body of literature has shown that forests provide nutritious foods in many low- and middle-income countries. Yet, there is limited evidence on the contributions from different types of forest and tree systems.

**Objectives:**

Here, we focus on individual trees and smaller forest patches outside established forest reserves as well as different forest management systems.

**Methods:**

We do so by combining novel high-resolution data on tree cover with 24-h dietary recall surveys from 465 women in Tanzania.

**Results:**

We show that people with more unclassified tree cover (i.e., individual trees and small forest patches) in their nearby surroundings have more adequate protein, iron, zinc, and vitamin A intakes. We also find that having a nearby forest under Participatory Forest Management (PFM) system is associated with higher adequacy levels of energy, iron, zinc and vitamin A. By contrast, tree cover within other types of forest (e.g., Government Forest Reserves and Government Forest Plantations) is not positively associated with people’s dietary quality.

**Conclusions:**

Our key finding is that having individual trees, smaller forest patches and/or forest under PFM in close proximity is more beneficial for people’s diets than other types of established forests. Our results highlight the nutritional importance of trees outside established forests and question the often-assumed benefits of forests if these are made inaccessible by social barriers (e.g., legislation). Finally, our results emphasize the need to distinguish between different forest management systems when studying forest-diet linkages.

**Supplementary Information:**

The online version contains supplementary material available at 10.1007/s10980-024-01961-6.

## Introduction

Recent literature has established positive linkages between forests and food and nutrition security in low- and middle-income countries, both based on large-scale datasets (Ickowitz et al. 2014; Galway et al. 2018; Rasolofoson et al. 2018; Den Braber et al. 2024) as well as site-specific case studies (Baudron et al. 2017; Cheek et al. 2022). There are four overarching pathways by which forests and trees can positively affect people’s food and nutrition security (Baudron et al. 2019b; Gergel et al. 2020): (1) The direct provision of food as forests often host numerous and nutrient-rich wild plants and animals that are consumed by local communities (Powell et al. 2013; Asprilla-Perea and Díaz-Puente 2019), (2) the provision of ecosystem services (e.g., soil protection, water provision, pollination, access to manure and biomass), which can improve the productivity of surrounding agricultural lands (Baudron et al. 2019a; Yang 2020), (3) the provision of fuelwood, which is vital for cooking and boiling water in many countries (Karki et al. 2018), and (4) income generation from sale of forest and tree products, which can facilitate the purchase of nutritious foods from markets (Miller et al. 2020).

Despite the well-established positive linkages between forests and people’s diets, little is known about (1) the potential contribution of trees outside of established forest reserves beyond agroforestry systems, which are well-studied (Babu and Rhoe 2002; Bostedt et al. 2016; Afentina et al. 2021), and (2) different types of forest management systems. We note that trees outside of established forest reserves differ from agroforestry as they are not limited to being located in or around farmland but include trees growing across all types of non-forest landscapes (e.g., urban settlements, roads, lakes). One potential reason behind the limited knowledge on the role of trees outside forests is that large-scale landscape studies tend to apply binary forest/non-forest classifications based on either moderate spatial resolution data (Johnson et al. 2013; Rasmussen et al. 2019) or larger political forest units (Kumeh et al. 2021). Consequently, most knowledge on tree-diet relationships comes from local case studies that examine the effects of agroforestry systems on people’s diets (Ghosh-Jerath et al. 2021; Jemal et al. 2021; Zahoor et al. 2021; Kulsum and Susandarini 2023). Such studies tend to find positive linkages between agroforestry and dietary quality (Jamnadass et al. 2013; Montagnini 2017; Dagar et al. 2020). For example, a cross-sectional study among 170 farmers in India estimated that a 1% increase in tree density and tree diversity on farms would increase people’s food consumption score (mean level: 28) by 0.2% point and 0.1% point, respectively (Singh et al. 2023). A recent review covering 36 publications on the linkages between tree-based farming systems and food and nutrition security in low- and middle-income countries found that trees located around farmland had generally positive effects on people’s diets, directly through provision of wild and cultivated foods, and indirectly through improved income opportunities (Vansant et al. 2022). Another review assessing 207 case studies from sub-Saharan Africa found that 68% of the studies indicated an increase in food availability due to the presence of trees on farms (Kuyah et al. 2016). Yet, a study among 399 farmers in six agroecological zones in Rwanda found that trees on farms mainly represented a safety net for the poorest households rather than an important contributor to overall food security (Ndoli et al. 2021). Also, there is mixed evidence on the effects of agroforestry on crop production with some studies pointing to the positive effects on yields (Baier et al. 2023) and soil quality (Kuyah et al. 2019), whereas other studies indicate that trees on farms may also be associated with lower yields of crops such as wheat (Khan et al. 2023).

Even though more than one quarter of Africa’s tree cover is found outside areas previously classified as forest (Reiner et al. 2023), the role of individual trees outside forests (beyond agroforestry) has long been overlooked due to a lack of high-resolution satellite imagery (Schnell et al. 2015). However, in the past few years progress has been made through the combination of new high-resolution satellite imagery and deep learning methods, which has enabled large-scale mapping of non-forest trees at the individual tree level. This includes the detection of 1.8 billion trees in West African Sahara and Sahel covering areas that had previously been perceived and categorized as bare dry lands or deserts (Brandt et al. 2020). Therefore, it is now possible—and needed—to examine more closely how trees outside of forests are related to people’s food and nutrition security in low- and middle-income countries.

The second knowledge gap that we aim to address is how different forest management systems can contribute to people’s diets, as management systems around forests can influence how people use forests and trees as a food source (Adhikari et al. 2016; Andrieu et al. 2019). For example, enforcement of environmental policies in Nepal combined with increased timber extraction has caused reductions in local livestock holdings due to lack of fodder resources, resulting in a worsening of people’s food security (Dhakal et al. 2011). The authors of this study suggested that policies could alternatively promote agroforestry systems combined with community-based forest management to gain both forest protection and better food security for local communities. This suggestion was later supported by another Nepalese study which, based on national survey data from 3064 rural households, found that households who used resources from community-based forests experienced higher levels of calorie adequacy compared to households using government-owned forests (Paudel 2018). Furthermore, a study from Tanzania assessed the effects of community-based forestry on wealth, food security and child health, and found improvements in household food security (measured by meals/day and fish and meat consumption) in areas with community-based forest management compared to areas without (Pailler et al. 2015). Also, a study in Cameroon reported that more than half of the community forest users were highly dependent on the forest resources, as these resources provided 61–100% of their income, food, energy and material needs (Ngang et al. 2018).

While these case studies from Nepal, Tanzania and Cameroon go beyond broad-scale studies that treat forests as a homogenous landscape feature, they tend to use broad food security metrics as opposed to more detailed measures of dietary quality. This absence of detailed dietary quality metrics was highlighted by a recent review of 30 publications on linkages between social forestry (the term was used by the authors to describe initiatives linking communities with sustainable forest management) and food security in Asia. The authors found that none of the publications examined how different forest management systems affect the dietary quality of local communities (Yahya et al. 2022). When examining the forest-diet relationship, it is important to move beyond crude measures of food security in favour of more detailed dietary quality metrics (where the data allows), as these measures provide more insight into the mechanisms driving the observed positive relationships.

In this study, we examined the effects of (1) unclassified tree cover (i.e., individual trees and small forest patches outside established forests) and (2) different types of forest management systems (e.g., Government Forest Reserve, Government Forest Plantation, Private Forest, Participatory Forest Management (PFM)) on people’s dietary quality, measured by macro- and micronutrient adequacy levels. By doing so, we demonstrate how different tree and forest systems can have varying effects on diets—and we thereby contribute to a more nuanced understanding of forest-tree-diet linkages.

## Material and methods

### Study sites

Tanzania is an appropriate country for studying the linkages between forests, trees, and people’s diets for a number of reasons. First, the country hosts several large bio-diverse forests (Capitani et al. 2019; Kacholi et al. 2015) and around 30% of the population live within 5 km of a forest (Newton et al. 2020). Second, case studies from different parts of the country have shown how communities rely on forest-based resources in their diet (Murray et al. 2001; Ceppi and Nielsen 2014; Kaya and Lyana 2014; Pollom et al. 2020). For example, a study in the North Uluguru and the West Usambara Mountains revealed that local communities consumed 114 different indigenous forest plant foods (Msuya et al. 2010). Another study among women living in close proximity to forests in the East Usambara Mountains identified 92 wild food species and found these wild foods to be an important source of vitamin A (31% of intake), vitamin C (20%), and iron (19%) for both women and children (Powell et al. 2013). Furthermore, deforestation in rural areas of Tanzania has been shown to reduce people’s fruit and vegetable consumption, with negative effects on vitamin A adequacy (Hall et al. 2022). Tanzania’s forests are also under increasing pressure from agricultural expansion and logging activities (Doggart et al. 2020). Finally, despite more than 20 years of sustained economic growth, culminating in its transition from low-income to lower middle-income status in 2020, the proportion of people suffering from severe food insecurity has increased from 21 to 26% between 2016 and 2022 (FAO et al. 2022). In addition, the number of people not able to afford a healthy diet increased from 49 to 52 million between 2017 and 2020, corresponding to 88% of the country’s population (FAO et al. 2022).

**Fig. 1:**
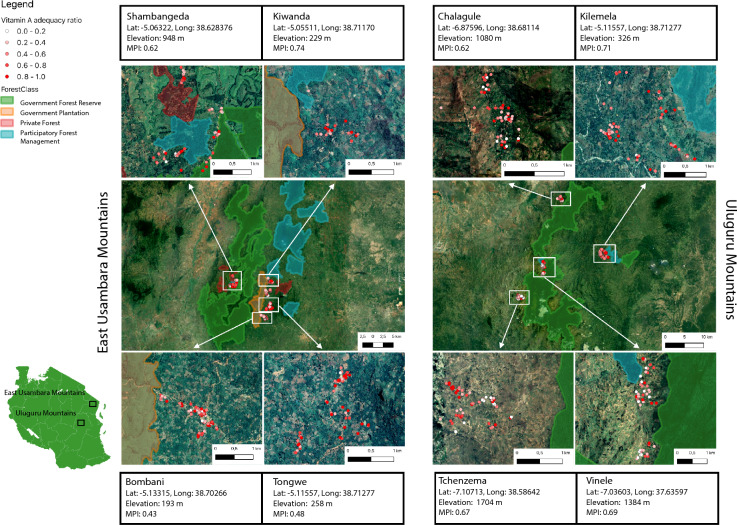
Forest management systems, position coordinates, elevation and mean Multidimensional Poverty Index (MPI) Living Standard dimension across the eight villages included in the study. The red dots represent the survey respondents’ homes and show variation in vitamin A adequacy levels within and across sites. The locations have been randomly displaced up to 300 m for confidentiality purposes

In this study, we collected data from eight villages in East Usambara Mountains and Uluguru Mountains in Tanzania from October to December 2021 (Fig. 1). The villages were selected to represent different forest management systems, while being relatively similar in terms of people’s living standards, agricultural practices, and climatic conditions.

Within each of the eight villages, we surveyed women with at least one child under the age of five years, since this group is particularly vulnerable to nutritional deficiencies (Lartey 2008). We selected 60 women from each village using a random stratified sampling technique. That is, every village consisted of four to eight hamlets, and we selected respondents from each hamlet proportional to its relative population size. For example, when 25% of the village’s total population lived in one hamlet, we would randomly select 25% (or 15 women) of our respondents from that hamlet.

### Forest management classification

We base our forest categories on Tanzania’s official forest classifications (United Republic of Tanzania, 1998, 2002). The country’s forests are grouped into the following categories of ownership: (1) *Central Government Forest Reserve*﻿—owned and managed by the central government, including both forest reserves and forest plantations), (2) *Local Authority Forest Reserve—*owned and managed by district authorities, (3) *Village Forest Reserve—*owned and managed by a village government, (4) *Private Forest* owned and managed by private companies, and (5) *forest patches in non-reserved forest land*—covering small tree plots less than 10 hectares, sometimes owned by the Central Government but most often with open access.

In addition, *Community Based Forest Management* (CBFM) takes place on village land. Villagers take full ownership and management responsibilities for the forest, and they also collect forest royalties from the sales of forest products and services. Finally, *Joint Forest Management* is based on a partnership between communities and the government and has typically been introduced in Central Government Forest Reserves that were previously under the management of the central government (United Republic of Tanzania 2008). The partnership means that communities are given more responsibilities in terms of managing the use of forest resources, while the central government continues to hold ownership (Mbwambo et al. 2012).

We regrouped these categories based on the actors managing the forest. *Village Forest Reserve*, *CBFM* and *Joint Forest Management* were combined into one category named *Participatory Forest Management* (PFM)*.* This regrouping is reasonable because (1) forest access was similar across these three types, and (2) forest management is given to local communities—yet with some differences in forest ownership (Khatun et al. 2015; Luswaga and Nuppenau 2020). Also, PFM is formally used as an umbrella term in Tanzania to cover the above-mentioned categories (United Republic of Tanzania 1998). Using QGIS and shapefiles showing official forest boundaries provided by the Central Government of Tanzania, we renamed and divided *Central Government Forest Reserves* into two groups: *Government Forest Reserves* and *Government Forest Plantations.* We renamed *forest patches in non-reserved forest land* to *unclassified tree cover* and expanded the category to include all trees outside of the above-mentioned forest categories, regardless of the plot size to also capture scattered trees in the landscape. *Private Forest* was maintained as a separate category. We did not include *Local Authority Forest Reserve* since this type of ownership was not present in any of our study sites.

### Forest and tree data

We collected GPS coordinates of the respondents’ homes, allowing us to measure the amount of tree cover in each respondent’s nearby surroundings. In this study, a tree is defined as a plant with a more or less permanent shoot system that is supported by a single trunk of wood (Mbuya et al. 1994). We used a Very High Resolution (VHR) map of African tree cover in 2019 (Reiner et al. 2023), which was created using a deep learning model to segment tree cover at the individual tree level, based on 3-m resolution PlanetScope. We spatially aggregated the binary tree cover data to extract the percentage tree cover in 2000-m radius buffer circles around each respondent’s house. We used a radius of 2000 m since this is the distance most wild foods are collected from people’s homes (Layton et al. 1991; Powell et al. 2011). We then overlaid this with shapefiles provided by the Tanzanian government on polygons of Government Forest Reserves, Government Forest Plantations, Private Forests, and PFMs. For each respondent, we thus obtained the percentage tree cover within each of the five forest/tree categories: Government Forest Reserve, Government Forest Plantation, Private Forest, PFM, and unclassified tree cover.

### Outcome variables

Most studies on forest-tree-diet linkages use food security metrics such as days without food, the household food insecurity access scale (Donn et al. 2016; Tata Ngome et al. 2019), or dietary diversity scores (Galway et al. 2018; Rasolofoson et al. 2018). Here, we go beyond these metrics by estimating people’s macro- and micronutrient intake, with our main outcome variables being people’s energy, protein, iron, zinc, and vitamin A adequacy. Nutrient adequacy ratios (NAR) were calculated from detailed dietary recall surveys, which aim to record every food item that the respondent has consumed within the past 24 hours. The 24-h dietary recalls were carried out twice within a week on two non-consecutive days to account for unusual dietary intakes (Gibson 2005). Quantities of each food item were estimated using local serving size aids (e.g., cups, plates, spoons) and photo aids.

We then estimated macro- and micronutrient contents of all reported food items using nutritional information from food composition tables (FCTs). A number of FCTs were used due to missing or incomplete nutrient information. We used data from the Tanzanian tables (Lukmanji and Hertzmark 2008) as much as possible. When data were missing, we sourced data from the FCTs for Kenya (FAO 2018a), Malawi (MAFOODS 2019), Zambia (NFNC 2009) and West Africa (Vincent et al. 2020)﻿—in that order.

Since all of our respondents were interviewed twice within one week, we were able to calculate the *usual* intake with a Multiple Source Method (MSM) (Tooze 2020). The methodology consists of three sequential steps: Initially, the probability of consuming a particular food on a given day is estimated for each individual. Subsequently, the usual amount of food intake on days when consumption occurs is estimated individually. Finally, the overall usual food intake across all days is computed by multiplying the probability of food consumption with the usual amount of food intake on consumption days (Harttig et al. 2011). We then calculated mean NAR by comparing the estimated nutrient intakes with average recommended nutrient intakes for energy (FAO et al. 2002), protein (WHO 2007), iron, zinc and vitamin A (WHO and FAO 2004). The adequacy ratios accounted for whether women were pregnant or breastfeeding. We note that our final adequacy ratios might be underestimated due to known issues with underreporting of certain food items, for example in cases where respondents eat from a shared bowl (Gibson 2005). Therefore, we interpret the calculated adequacy levels as relative values between respondents rather than total values to be compared with national or international averages.

We calculated dietary diversity scores (DDS) given that more diverse diets are a good proxy for micronutrient intake and overall dietary quality (Kennedy et al. 2007; Verger et al. 2019). To measure dietary diversity, we used the Minimum Dietary Diversity Score for Women (MDD-W), which categorizes foods into ten groups: (1) Grains, white roots and tubers, and plantains, (2) pulses (beans, peas and lentils), (3) nuts and seeds, (4) dairy, (5) meat, poultry and fish, (6) eggs, (7) dark green leafy vegetables, (8) other vitamin A-rich fruits and vegetables, (9) other vegetables, and (10) other fruits (FAO 2021; FAO and FHI 360 2016).

In addition to calculating DDS, we focused specifically on the consumption of each of the six most nutritionally important food groups (‘grains, white roots and tubers, and plantains’, ‘pulses’, ‘meat, poultry and fish’, ‘dark green leafy vegetables’, ‘other vitamin A-rich fruits and vegetables’, and ‘other fruits’). Together, these six groups represent 99% of respondents’ nutrient intake (i.e., for protein, iron, zinc, and vitamin A) (Fig. S2).

### Covariates

We controlled for a number of variables known to affect people’s diets and thus confound the relationship between forests, trees, and diets. We controlled for agricultural practices (i.e., total crop count, homegarden presence, tropical livestock units (TLU))—as more diverse crop and/or livestock production can lead to better overall dietary quality (Ali and Khan 2013; Jones 2017; Headey et al. 2018; Sibhatu and Qaim 2018; Christian et al. 2019). We calculated TLU using conversion factors for each livestock owned by the household according to FAO’s guidelines (FAO 2018b). We also controlled for individual and household characteristics known to affect diets, including age (Malapit et al. 2015), education level measured as years of schooling (Torheim et al. 2004), living standards, region, and household size (Workicho et al. 2016; Powell et al. 2017). To assess living standards, we used the Multidimensional Poverty Index (MPI) Living Standard dimension, ranging from 1 (deprived) to 0 (not deprived) and based on six indicators; cooking fuel, sanitation, drinking water, electricity, housing, and assets (Alkire et al. 2021). We used the distance to the nearest road from the household (based on respondents’ estimated walking time) as a proxy for market access, which is known to influence people’s consumption of specific foods (Ickowitz et al. 2019). We used distance to the nearest road rather than other variables such as distance to the nearest market as local people had different perceptions of market definitions (e.g., minor stand by the road, permanent market, travelling market). Finally, when using one of the five tree or forest categories as the ‘treatment’ variable (e.g., unclassified tree cover), we controlled for the other four categories (e.g., Government Forest Reserve, Government Forest Plantation, Private Forest, and PFM). Table S1 provides an overview of all covariates.

### Statistical approach

We tested whether tree cover (%) within our five tree and forest categories was associated with people’s dietary adequacy and dietary diversity. We employed Covariate Balancing Generalized Propensity Score (CBGPS) matching, which is a quasi-experimental technique. CBGPS was chosen because it is robust to model misspecifications and applicable in the case of a continuous treatment (Imai and Ratkovic 2014). The weights produced by CBGPS minimize the correlation between treatment and observable pre-treatment covariates when included in the subsequent regression models. This reduces the dependence (endogeneity) between treatment assignment and outcome given covariates. If not addressed, this dependence may lead to biased estimates of the effects of tree cover on people’s dietary quality. CBGPS extends traditional propensity score methods used for binary treatments by creating inverse propensity score weights (Fong et al. 2018). To calculate CBGPS weights, we used the control variables mentioned earlier as pre-treatment variables: Crop count, homegarden presence, TLU, age and educational level of the respondent, MPI living standards, household size, region, distance to nearest road and the remaining four forest and tree categories not acting as the treatment. We used the CBPS package (Fong et al. 2022) in R (version 4.3.2) to perform the matching analyses. Correlations between treatment (tree cover inside Government Forest Reserve, Government Forest Plantation, Private Forest, and PFM and unclassified tree cover included one by one controlling for the other types) and covariates were sufficiently reduced after matching (Fig. S1). When using NAR as the outcome variable (i.e., adequacy levels for energy, protein, iron, zinc and vitamin A), we fitted a linear model using the CBGPS weights, with tree cover within the five different types of tree and forest systems as the key predictor of interest. When using the consumption of the six focus food groups (grams of unique food items consumed within each food group), we used the same model specification. When using DDS as the outcome, we applied a quasipoisson generalized linear model to account for the non-continuous categorical outcome variable (Warton et al. 2016). We checked for overdispersion using the ‘dispersiontest’ function in the AER package in R, but found no overdispersion in our models and therefore did not use the negative binomial distribution (Kleiber et al. 2020). Finally, we used the sandwich package to compute cluster-robust standard errors at the village level to adjust for the lack of independence of households within the same village (Zeileis et al. 2020).

We used both a pairwise correlation matrix as well as the variance inflation factor (VIF) to assess potential collinearity among independent variables included in our models. All correlation coefficients were < 0.5 and VIF did not exceed a value of 5. Lastly, to check the robustness of our results, we re-ran all models using a 1000-m radius instead of 2000-m radius (Table S2).

## Results

**Fig. 2:**
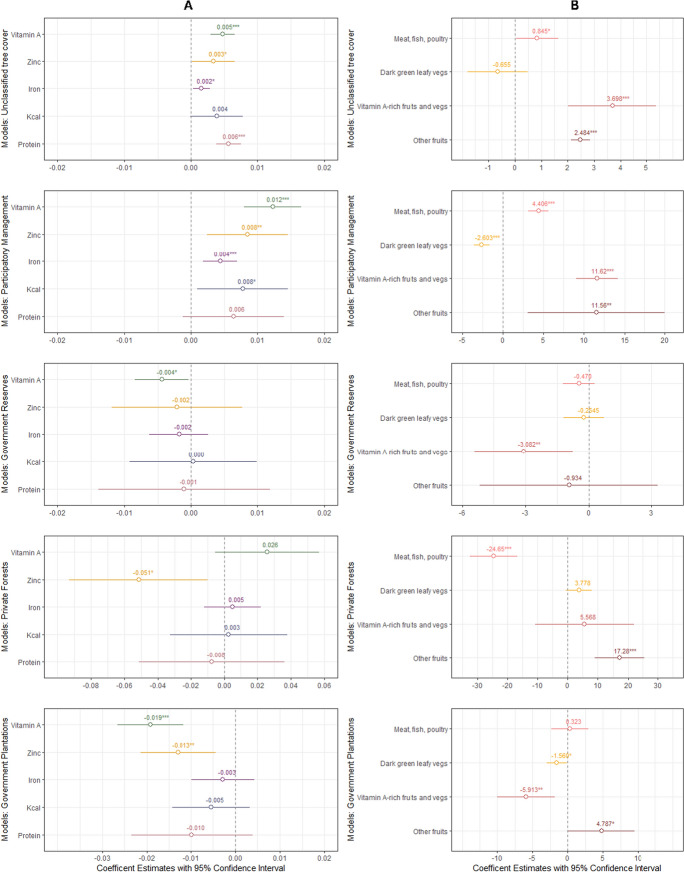
Post-matching results for how tree cover within five different types of tree and forest management systems is associated with people’s **A** macro- and micronutrient adequacy, and **B** intake of four key food groups. Results are not shown for the two food groups ‘grains, white roots and tubers, and plantains’ and ‘pulses’ as no significant results were found. P-values: * < 0.05, ** < 0.01, *** < 0.001. N = 465

Our study has two main findings: (1) People living in areas with more unclassified tree cover (covering individual trees and forest patches) appear to have higher adequacy levels of protein, iron, zinc, and vitamin A. (2) People living in areas with greater tree cover within PFM appear to have higher adequacy levels of energy, iron, zinc, and vitamin A (Fig. 2; Table S1).

## Positive associations between unclassified tree cover and dietary quality

We found that the amount of unclassified tree cover is positively associated with people’s adequacy levels of protein and all three micronutrients. That is, a 1% increase in unclassified tree cover translates into higher adequacy levels of 0.6% for protein (p < 0.001), 0.2% for iron (p < 0.05), 0.3% for zinc (p < 0.05), and 0.5% for vitamin A (p < 0.001) (Fig. 2A).

With the mean unclassified tree cover being 28.9%, an increase from no tree cover to this level would translate into 16.4%, 4.7%, 9.8%, and 14% higher adequacy levels of protein, iron, zinc, and vitamin A, respectively. Although such increases may not appear substantial, they are notable given that dietary adequacy is very low in our study area. For example, only 22% of our respondents meet protein requirements, no respondents meet iron requirements, 4% meet zinc requirements, and 14% meet vitamin A requirements.

However, such translation should be considered with caution since it assumes a continuous linear effect between increases in unclassified tree cover and people’s nutrient adequacy. Recent studies have shown how forests and trees can be linked to diets in non-linear ways (Friant et al. 2019; Rasmussen et al. 2019; Kumeh et al. 2022). Likewise, the potential underestimation of our respondents’ nutrient adequacy levels merits caution when interpreting these estimates.

**Fig. 3:**
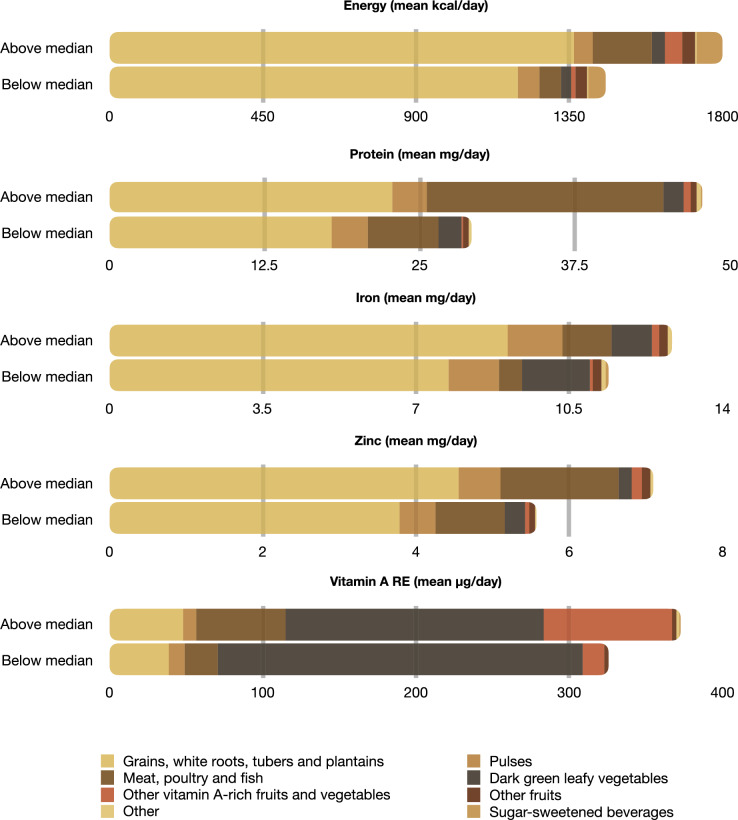
Share of energy, protein and micronutrients coming from the different MDD-W food groups, broken down into respondents living in areas with above vs below median unclassified tree cover. We have merged ‘nuts and seeds’, ‘dairy’, ‘eggs’ and ‘other vegetables’ into ‘other’ because these food groups contributed less than 1% of total nutrient intake. The category ‘sugar sweetened beverages’ was added to the figure as it contributed 4.5% and 3.5% of energy intake for respondents living in areas with above and below median unclassified tree cover, respectively. N = 465

Along with effects on nutrient adequacy levels, we also examined the effects of unclassified tree cover on people’s intake of six key food groups. We found a positive association with the intake of three food groups: ‘meat, poultry and fish’ (p < 0.05), ‘other vitamin A-rich fruits and vegetables’ (p < 0.001) and ‘other fruits’ (p < 0.001) (Fig. 2B). Respondents with above median levels of unclassified tree cover on average consumed 111 g per day of ‘meat, poultry and fish’ compared to only 40 g for those respondents with below median tree cover (Table 1; Fig. 3). Given that 91% of the total amount of ‘meat, poultry and fish’ consumed was fish (Fig. S2), it is likely that this was the main driver of higher protein, iron and zinc intakes for those respondents living in areas with higher unclassified tree cover. This is in line with other studies that have documented the importance of fish consumption for dietary quality in East Africa (Wessels et al. 2023). Furthermore, it is likely that higher intakes of ‘other vitamin A-rich fruits and vegetables’ (especially mangos and papayas (Fig. S2)) explain the observed positive associations between unclassified tree cover and the higher adequacy levels of vitamin A.

**Table 1:** Mean values of covariates for respondents living in areas with above vs below median levels of unclassified tree cover and in areas with vs without PFM within a 2000-m radius

	Mean (SD)			
Variables	Unclassified tree cover		Participatory forest management (PFM)	
	Above median	Below median	With	Without
Age	30.19 (7.44)	29.16 (8.11)	29.06 (7.79)	30.06 (7.78)
Household size	5.24 (1.84)	5.13 (2)	5.21 (2.07)	5.17 (1.82)
Number of crops cultivated	5.07 (2.78)	4.6 (2.63)	4.87 (2.73)	4.81 (2.71)
Tropical livestock unit (TLU)	0.26 (0.85)	0.26 (0.5)	0.29 (0.8)	0.24 (0.63)
Distance to nearest road (minutes walking)	95.7 (163.63)	68.84 (88.92)	122.44 (180.44)	57.24 (81.01)
Multidimensional poverty index (MPI)	0.63 (0.2)	0.62 (0.16)	0.62 (0.18)	0.63 (0.18)
Energy adequacy ratio (%)	0.77 (0.17)	0.65 (0.22)	0.71 (0.19)	0.71 (0.21)
Protein adequacy ratio (%)	0.72 (0.26)	0.53 (0.31)	0.60 (0.28)	0.64 (0.31)
Iron adequacy ratio (%)	0.44 (0.12)	0.39 (0.14)	0.42 (0.13)	0.41 (0.13)
Zinc adequacy ratio (%)	0.6 (0.2)	0.48 (0.22)	0.54 (0.22)	0.54 (0.22)
Vitamin A adequacy ratio (%)	0.62 (0.25)	0.56 (0.25)	0.63 (0.26)	0.56 (0.24)
DDS	3.69 (1.45)	3.97 (1.36)	4.1 (1.5)	3.67 (1.36)
Grains, white roots and tubers, and plantains (mean g/day)	1044.8 (406.25)	908.9 (432)	997.06 (427.27)	964.46 (422.76)
Pulses (mean g/day)	54.43 (70.56)	55.33 (78.37)	50.75 (70.76)	57.46 (76.72)
Meat, poultry and fish (mean g/day)	110.71 (115.5)	40.12 (68.5)	73.36 (97.25)	76.82 (103.84)
Dark green leafy vegetables (mean g/day)	48.89 (56.5)	57.29 (76.21)	43.23 (48.52)	59.23 (75.91)
Other vitamin A-rich fruits and vegetables (mean g/day)	101.81 (197.07)	23.31 (81.97)	99.45 (182.17)	39.66 (132.21)
Other fruits (mean g/day)	45.81 (148.78)	51.76 (199.27)	55.65 (199.2)	44.49 (159.37)

N = 465

## Positive associations between participatory forest management and dietary quality

We also found that the extent of tree cover classified as PFM is positively associated with higher adequacy levels of energy, iron, zinc, and vitamin A as well as higher dietary diversity scores (Fig. S3). That is, a 1% increase in tree cover within PFM translates into increases of nutrient adequacy levels of 0.7% for energy (p < 0.05), 0.4% for iron (p < 0.001), 0.8% for zinc (p < 0.01), and 1.3% for vitamin A (p < 0.001) (Fig. 2A). This is likely driven by higher consumption of fish, other vitamin A-rich fruits and vegetables, and other fruits, as the consumption of these food groups was also significantly positively associated with tree cover within PFM (Fig. 2B).

By contrast, tree cover within Government Forest Plantations was negatively associated with adequacy levels of zinc (− 1.3%, p < 0.05) and vitamin A (− 1.9%, p < 0.001). Similarly, we found negative associations between tree cover within Government Forest Reserves and vitamin A (− 0.4%, p < 0.05) and Private Forests and people’s adequacy level of zinc (− 5.1%, p < 0.05). These results might be explained by lower consumption of vitamin A-rich fruits and vegetables and dark green leafy vegetables by people living in areas with more tree cover within Government Forest Plantation (− 5.9%, p < 0.01, − 1.6%, p < 0.05, respectively). Likewise, people living in areas with more tree cover within Government Forest Reserves had lower consumption of vitamin A-rich fruits and vegetables (− 3.1%, p < 0.05).

## Discussion

### Trees and forest patches outside forests are beneficial for dietary quality

Our findings suggest that trees and small forest patches outside of established forest reserves can improve people’s nutrient adequacy. These findings demonstrate the importance of moving beyond forest/non-forest dichotomies, which have been a common approach in the forest-diet literature (e.g. Johnson et al. 2013; Galway et al. 2018). Also, the existing bulk of research on relationships between tree-based farming systems and food and nutrition security (Vansant et al. 2022) tends to focus on trees in croplands and thereby dismisses the potential contributions from individual trees in fallows, pasture, around settlements or along roads, lakes and rivers. By using VHR satellite data, we were able to include trees that would not be accounted for otherwise—both on farms and scattered trees outside of forests. While existing studies attending to on-farm trees often rely on self-reported counts or time-consuming field measurements, our method can be extrapolated and potentially up-scaled to cover even greater areas.

However, we were not able to distinguish between different types of trees (e.g., timber trees vs fruit trees), which limits the ability to tease apart causal mechanisms between tree cover and dietary quality. For example, we found a positive significant relationship between unclassified tree cover and people’s vitamin A adequacy levels as well as their consumption of vitamin A-rich fruits and vegetables (Fig. 2 and Table S1). These relationships indicate that people living in areas with higher tree cover might be consuming more vitamin A-rich fruits harvested from trees, such as mango and papaya. When looking at where people source their vitamin A-rich fruits and vegetables, we observe that people living in areas with above median tree cover source a higher proportion of this food group from the wild (4.5% as compared to 0% for people living in areas with below median tree cover) as well as from cultivated fields (61% as compared to 52%) (Table 2). Such explanation would be in line with other studies that have established a positive role of fruit trees for diets (Jamnadass et al. 2011; Bostedt et al. 2016; Mathewos et al. 2018; Omotayo and Aremu 2020; Kulsum and Susandarini 2023). For example, a study from Ethiopia found that growing fruit trees was positively associated with higher dietary diversity among women and young children in the households (Desalegn and Jagiso 2020).

**Table 2:** The share of six focus food groups coming from different sources (cultivated, purchased or wild) among respondents living with above vs below median unclassified tree cover within a 2000-m radius

	Source (% of food items)					
Food group	Cultivated		Purchased		Wild	
	Above median	Below median	Above median	Below median	Above median	Below median
Grains, white roots and tubers, and plantains	33.5	30.2	65.8	68.4	0.6	1.4
Pulses	9.8	19.9	89.9	79.5	0.3	0.6
Meat, poultry and fish	0.2	0.7	97.9	97.9	1.8	1.4
Dark green leafy vegetables	30.9	35.4	51.3	45.6	17.8	19.0
Other vitamin A-rich fruits and vegetables	60.9	52.4	34.5	47.6	4.5	0.0
Other fruits	45.1	29.4	51.0	64.7	3.9	5.9

N = 465

We also found a positive association between unclassified tree cover and adequacy levels of protein, iron, and zinc. This may be explained by higher fish consumption among people living in areas with high tree cover. Positive associations between tree cover and fish consumption have also been documented in both Indonesia (Ickowitz et al. 2023) and Nigeria (Lo et al. 2019), suggesting that trees provide ecosystem services that enhance the availability of fish stocks. While we do observe a marginal higher proportion of fish being sourced from the wild among people living in areas with higher tree cover (1.8% as compared to 1.4% among people living in areas with below median tree cover), most of the consumed fish is purchased from the market rather than caught in local rivers and lakes (Table 2). Nevertheless, the nutritional importance of fish in East Africa is notable (Béné et al. 2016). It has been estimated that utilizing the entire amount of the potential sustainable catch of Silver cyprinid (small pelagic fish) in Lake Victoria would provide a sufficient daily source of vitamin B12, calcium, zinc and iron to approximately 33 million people in the region (Wessels et al. 2023). Twenty-five percent of our respondents consumed less than 100 g of fish relish per day. Thus, a relatively small increase in fish consumption may be a promising avenue to increase nutrient adequacy levels.

## Linkages between forest management systems and dietary quality

It is well established that the type of forest management system in place matters for the type and quantity of products that people can harvest from the forest—and thereby influence the potential of forests to alleviate poverty (Miller et al. 2020). Yet, the role of forest management systems in relation to dietary quality has been somewhat overlooked, especially in quantitative studies. Here, we find substantial variations across different forest management systems, with positive effects seen in PFM systems and negative effects of other forest management systems (Government Forest Reserves and Government Forest Plantations).

These negative effects on people’s diets exemplify how forest conservation initiatives and profit-oriented forestry might have unintended consequences for food and nutrition security when people’s access to these forests is restricted. For example, the respondents in our study sites were only allowed to enter the Government Forest Reserves and Government Forest Plantations once a week to collect fuelwood and wild plants. When entering the Government Forest Reserves, they were not allowed to bring a machete, which made the dense part of the forests impenetrable. Also, the Government Forest Plantations were dominated by one exotic teak tree species (*Tectona grandis*) with low levels of biodiversity and relatively few wild foods to find. Multiple studies from various parts of the world have described how forest conservation can lead to negative social outcomes if local people are not appropriately compensated or included in the management regimes (Blaney et al. 2009; Ibarra et al. 2011; Sylvester et al. 2016; Nakamura and Hanazaki 2017; Campbell et al. 2021). For example, Hall et al. (2014) assessed both ecological and livelihood consequences of the newly established Derema Forest, a large protected forest corridor in East Usambara Mountains. Two years after establishment, the area appeared to succeed in terms of functioning as an ecologically important corridor but failed to mitigate livelihood losses especially for the poorest people (Hall et al. 2014). Likewise, forest conservation in Oaxaca, Mexico was perceived to make indigenous communities more food insecure as local community members found a decrease in subsistence crop yields, land available for agriculture and shortened fallow cycles to be a result of implemented conservation policies (Ibarra et al. 2011). More recently, a study from Southwestern Ghana suggested that forest conservation initiatives should be combined with so-called ‘food security corridors’ in degraded forest-fringes to ensure that local populations benefit from both forests and cultivated resources—which in turn can reduce exploitation of the inner forest reserve (Kumeh et al. 2022). Yet, we note that previous studies have also shown how mixed plantations and private forests can provide a variety of beneficial ecosystem services, including local food provision (Liu et al. 2018). For example, a study from the Congo Basin examined land use competition between timber concessions and fruit trees harvested by local communities and found that both interests could co-exist as long as timber harvesting only targeted the largest trees and allowed appropriate minimum distances between the remaining trees to ensure gene flow for future forest regeneration (Snook et al. 2015).

It is also important to emphasize that PFM is an umbrella term that covers different sub-management systems (e.g., Joint Forest Management, Community Forest Management and Village Forest Management). Although we found it reasonable to group these into one category based on similarities in terms of access to resources, these sub-systems may differ in other aspects that may affect dietary quality. For example, a study from Tanzania comparing Community Forest Management and Joint Forest Management found that the level of participation was higher among communities with Joint Forest management (Luswaga and Nuppenau 2020), yet the study did not measure differences in resource use. Also, PFM is not always found to have the anticipated positive effects on local livelihoods, and potential co-benefits are most often dependent on site-specific contextual factors (Duguma et al. 2018; Hajjar and Oldekop 2018). For example, participatory forest initiatives in Nepal have been centred around timber extraction and biodiversity conservation, while disregarding food security outcomes for local people (Khatri et al. 2017). In other words, while the results of this study suggest that the inclusion of local communities in forest management systems is more likely to produce dietary benefits, as compared to more exclusive and inaccessible forests management systems, PFM should not be perceived as a panacea to improve food and nutrition security.

## Policy implications and future research directions

Our findings have policy relevance in terms of future strategies for improving local people’s food and nutrition security, particularly in rural areas of low- and middle-income countries. In particular, our findings have two key policy implications:

1. Decision-makers should support initiatives towards multi-functional and nutrition-rich landscapes through the promotion of trees and forest patches outside established forest reserves and in near surroundings of the targeted populations.
2. Because we show positive effects of PFM systems on local people’s diets, but negative effects of other forest management systems, decision-makers should attend to sustainable food extraction from community-based forests (e.g., apiculture and foraging of wild foods and medical plants).

Moreover, our study allows us to point to a number of directions for future research. Firstly, future research on linkages between forests, trees and dietary quality should move beyond dichotomies of forest versus non-forest. Trees grow not only in established forest blocks or on farmlands but are scattered across the landscape and are present along roads, rivers, and lakes. Secondly, while we have shown the potential importance of these scattered trees (not constituting a forest) in Tanzania, more work is needed to examine whether these relationships hold in other countries and contexts. Thirdly, our approach does not allow us to tease apart the dietary contributions from different tree species. Future research efforts would benefit from identification of and distinction between different tree species and their effect on people’s diets.

## Supplementary Information

Below is the link to the electronic supplementary material.

Supplementary file1 (DOCX 1038 KB)

## Data Availability

The data are available from the authors upon reasonable request and with the permission of University of Copenhagen.
