# Supplementary material for: The importance of different forest management systems for people’s dietary quality in Tanzania
Source: Landsc Ecol. 2024 Sep 11;39(9):176. doi: 10.1007/s10980-024-01961-6 (PMC11390844; doi:10.1007/s10980-024-01961-6)
Supplement: Supplementary file 1 — Supplementary file1 (DOCX 1038 KB) [file 10980_2024_1961_MOESM1_ESM.docx]

**
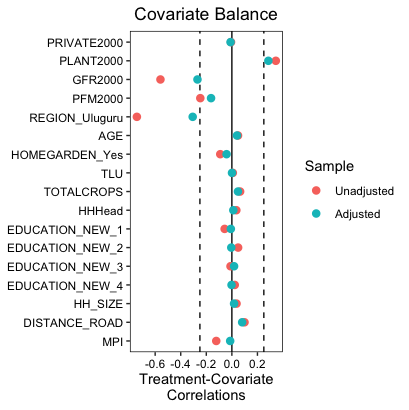
**

A B


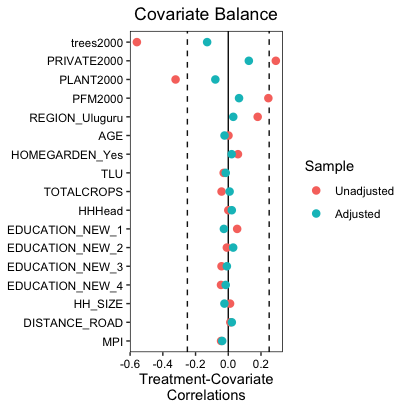

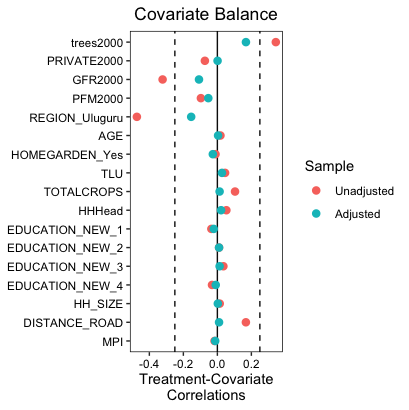


C D


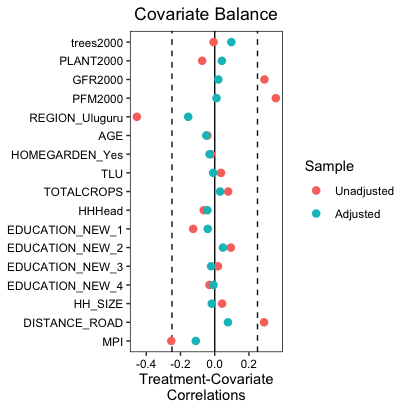


E

**Fig. S1** Balance of covariate before and after CBGPS matching for five models with the following ‘treatment’ variables: A) unclassified tree cover in 2000m radius (trees2000), B) tree cover within Participatory Forest Management within 2000m (PFM2000), C) tree cover within Government Forest Reserves within 2000m (GFR2000), D) tree cover within Government Plantations within 2000m (PLANT2000) and E) tree cover within Private Forest within 2000m (PRIVATE2000)*.* Absolute Pearson correlations for confounding covariates before matching (red circles) and after matching (blue circle).

** Fig. S2** The most prevalent foods consumed from each food group. The six food groups are the food groups with the largest nutrient contributions. N=465 women.


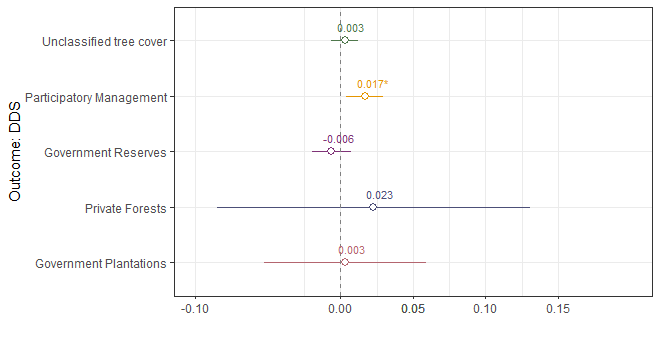


**Fig. S3** Post-matching results for how tree cover within five different types of tree and forest management systems is associated with people’s dietary diversity scores (DDS). p-values: *<0.05. N=465.

**Table S1** Model coefficients from five different treatments: Tree cover (%) classified as 1) Unclassified tree cover (TREES), 2) Participatory Forest Management (PFM), 3) Government Forest Reserve (GFR), 4) Government Plantations (PLANT), and 5) Private Forests (PRIVATE). For each treatment variable, we ran 12 models with the following outcomes: zinc adequacy, vitamin A adequacy, iron adequacy, Kcal adequacy, protein adequacy, DDS, consumption (in grams/person/day) of the following six food groups: ‘Grains, white roots and tubers, and plantains’, ‘pulses (beans, peas and lentils)’, ‘meat, poultry and fish’, ‘dark green leafy vegetables’,’ other vitamin A-rich fruits and vegetables’, and ‘other fruits’. Radius around each household: 2000m.

| Treatment | Covariates | Model | Outcome | Estimate | Std. Error | t value | P-value |
| --- | --- | --- | --- | --- | --- | --- | --- |
| trees2000 | trees2000 + PRIVATE2000+ PLANT2000 + GFR2000 + PFM2000 + Region + Homegarden + Age + Number of crops + Tropical Livestock Unit (TLU) + Head of household (man/woman) + Educational level + Household size + Distance to nearest road + Multidimensional Poverty Index (MPI) | CBPS (lm) | Zinc | 0.00340592 | 0.00162874 | 2.09113463 | 0.03708641 |
|  |  |  | Vitamin A | 0.0048051 | 0.00091736 | 5.23793627 | 2.5178E-07 |
|  |  |  | Iron | 0.00162067 | 0.00063277 | 2.56123195 | 0.01076107 |
|  |  |  | Kcal | 0.00387731 | 0.0019983 | 1.94030198 | 0.05297861 |
|  |  |  | Protein | 0.00568226 | 0.00094512 | 6.01222122 | 3.8351E-09 |
| PFM2000 | trees2000 + PRIVATE2000+ PLANT2000 + GFR2000 + Region + Homegarden + Age + Number of crops + TLU + Head of household (man/woman) + Educational level + Household size + Distance to nearest road + MPI | CBPS (lm) | Zinc | 0.0084716 | 0.00307937 | 2.75108247 | 0.00618391 |
|  |  |  | Vitamin A | 0.01226188 | 0.00217335 | 5.64193398 | 3.0072E-08 |
|  |  |  | Iron | 0.00439556 | 0.001302 | 3.3759999 | 0.00080044 |
|  |  |  | Kcal | 0.00777331 | 0.00344836 | 2.25420429 | 0.02467227 |
|  |  |  | Protein | 0.00639292 | 0.00384452 | 1.66286793 | 0.09704776 |
| GFR2000 | trees2000 + PRIVATE2000+ PLANT2000 + PFM2000 + Region + Homegarden + Age + Number of crops + TLU + Head of household (man/woman) + Educational level + Household size + Distance to nearest road + MPI | CBPS (lm) | Zinc | -0.0021037 | 0.00497649 | -0.4227341 | 0.67269488 |
|  |  |  | Vitamin A | -0.0044015 | 0.00206128 | -2.1353043 | 0.03328532 |
|  |  |  | Iron | -0.0018306 | 0.00227513 | -0.8046261 | 0.42146806 |
|  |  |  | Kcal | 0.00034437 | 0.0048513 | 0.07098562 | 0.94344131 |
|  |  |  | Protein | -0.0010181 | 0.00654134 | -0.1556426 | 0.8763858 |
| PLANT2000 | trees2000 + PRIVATE2000+ GFR2000 + PFM2000 + Region + Homegarden + Age + Number of crops + TLU + Head of household (man/woman) + Educational level + Household size + Distance to nearest road + MPI | CBPS (lm) | Zinc | -0.0129042 | 0.00430151 | -2.9999182 | 0.00285318 |
|  |  |  | Vitamin A | -0.0191297 | 0.00379931 | -5.0350409 | 6.9696E-07 |
|  |  |  | Iron | -0.0027762 | 0.00359131 | -0.7730347 | 0.43991502 |
|  |  |  | Kcal | -0.0054865 | 0.00443732 | -1.236448 | 0.2169487 |
|  |  |  | Protein | -0.0097915 | 0.00694177 | -1.4105122 | 0.15909188 |
| PRIVATE2000 | trees2000 + PLANT2000 + GFR2000 + PFM2000 + Region + Homegarden + Age + Number of crops + TLU + Head of household (man/woman) + Educational level + Household size + Distance to nearest road + MPI | CBPS (lm) | Zinc | -0.0514772 | 0.02120858 | -2.4271889 | 0.01561497 |
|  |  |  | Vitamin A | 0.02561115 | 0.01583518 | 1.61735767 | 0.1065145 |
|  |  |  | Iron | 0.00498036 | 0.00860727 | 0.57862238 | 0.56313872 |
|  |  |  | Kcal | 0.00251702 | 0.01798695 | 0.13993598 | 0.88877432 |
|  |  |  | Protein | -0.0075397 | 0.02228192 | -0.3383796 | 0.73523779 |
| TREES2000 | trees2000 + PRIVATE2000+ PLANT2000 + GFR2000 + PFM2000 + Region + Homegarden + Age + Number of crops + TLU + Head of household (man/woman) + Educational level + Household size + Distance to nearest road + MPI | CBPS (glm, family=quasipoisson) | DDS | 0.00285158 | 0.00465053 | 0.61317461 | 0.53976089 |
|  |  | CBPS (lm) | Grains, white roots and tubers, and plantains | 5.77100213 | 2.76784247 | 2.08501827 | 0.03764094 |
|  |  |  | Pulses | 0.62485443 | 0.59608224 | 1.04826883 | 0.29508762 |
|  |  |  | Meat, poultry and fish | 0.84538555 | 0.40850652 | 2.06945425 | 0.03908403 |
|  |  |  | Dark green leafy vegetables | -0.654593 | 0.57927124 | -1.1300285 | 0.25907707 |
|  |  |  | Other vitamin A-rich fruits and vegetables | 3.69801593 | 0.85333624 | 4.33359764 | 1.8177E-05 |
|  |  |  | Other fruits | 2.48375088 | 0.18390357 | 13.5057244 | 4.8325E-35 |
| PFM2000 | trees2000 + PRIVATE2000+ PLANT2000 + GFR2000 + Region + Homegarden + Age + Number of crops + TLU + Head of household (man/woman) + Educational level + Household size + Distance to nearest road + MPI | CBPS (glm, family=quasipoisson) | DDS | 0.01671361 | 0.00649916 | 2.57165556 | 0.01012135 |
|  |  | CBPS (lm) | Grains, white roots and tubers, and plantains | 5.27733009 | 5.59867313 | 0.94260371 | 0.34639855 |
|  |  |  | Pulses | 0.67228391 | 0.98956542 | 0.67937288 | 0.49725724 |
|  |  |  | Meat, poultry and fish | 4.40552956 | 0.62838351 | 7.01089298 | 8.9107E-12 |
|  |  |  | Dark green leafy vegetables | -2.6032067 | 0.48965713 | -5.3163869 | 1.6834E-07 |
|  |  |  | Other vitamin A-rich fruits and vegetables | 11.617518 | 1.30441284 | 8.90631986 | 1.3814E-17 |
|  |  |  | Other fruits | 11.5579416 | 4.30142695 | 2.68700171 | 0.00748154 |
| GFR2000 | trees2000 + PRIVATE2000+ PLANT2000 + PFM2000 + Region + Homegarden + Age + Number of crops + TLU + Head of household (man/woman) + Educational level + Household size + Distance to nearest road + MPI | CBPS (glm, family=quasipoisson) | DDS | -0.0062123 | 0.00688974 | -0.9016722 | 0.367231 |
|  |  | CBPS (lm) | Grains, white roots and tubers, and plantains | 0.2618524 | 5.48123213 | 0.04777254 | 0.9619191 |
|  |  |  | Pulses | 0.13957373 | 1.1389341 | 0.12254768 | 0.90252099 |
|  |  |  | Meat, poultry and fish | -0.4667786 | 0.38525826 | -1.2115993 | 0.22631314 |
|  |  |  | Dark green leafy vegetables | -0.2447897 | 0.49180256 | -0.4977398 | 0.61891499 |
|  |  |  | Other vitamin A-rich fruits and vegetables | -3.08199 | 1.17906331 | -2.6139309 | 0.00925624 |
|  |  |  | Other fruits | -0.9342196 | 2.15512641 | -0.4334871 | 0.66487238 |
| PLANT2000 | trees2000 + PRIVATE2000+ GFR2000 + PFM2000 + Region + Homegarden + Age + Number of crops + TLU + Head of household (man/woman) + Educational level + Household size + Distance to nearest road + MPI | CBPS (glm, family=quasipoisson) | DDS | 0.00308889 | 0.02844712 | 0.10858359 | 0.91353277 |
|  |  | CBPS (lm) | Grains, white roots and tubers, and plantains | -13.432613 | 3.32557553 | -4.0391845 | 6.324E-05 |
|  |  |  | Pulses | -0.1344755 | 2.9414165 | -0.0457179 | 0.96355572 |
|  |  |  | Meat, poultry and fish | 0.32298939 | 1.32706873 | 0.24338558 | 0.80781963 |
|  |  |  | Dark green leafy vegetables | -1.5604536 | 0.73763101 | -2.1154935 | 0.03494669 |
|  |  |  | Other vitamin A-rich fruits and vegetables | -5.9132381 | 2.09501235 | -2.8225314 | 0.00497962 |
|  |  |  | Other fruits | 4.78741538 | 2.39983887 | 1.99489034 | 0.04666841 |
| PRIVATE2000 | trees2000 + PLANT2000 + GFR2000 + PFM2000 + Region + Homegarden + Age + Number of crops + TLU + Head of household (man/woman) + Educational level + Household size + Distance to nearest road + MPI | CBPS (glm, family=quasipoisson) | DDS | 0.02259628 | 0.0550254 | 0.41065188 | 0.68132782 |
|  |  | CBPS (lm) | Grains, white roots and tubers, and plantains | 50.3509739 | 26.9066048 | 1.87132395 | 0.06196056 |
|  |  |  | Pulses | -1.0583263 | 4.86765982 | -0.2174199 | 0.82798143 |
|  |  |  | Meat, poultry and fish | -24.646098 | 3.99098416 | -6.1754437 | 1.4966E-09 |
|  |  |  | Dark green leafy vegetables | 3.77767532 | 2.19144239 | 1.72383053 | 0.08543769 |
|  |  |  | Other vitamin A-rich fruits and vegetables | 5.56839098 | 8.33230468 | 0.66828941 | 0.5042977 |
|  |  |  | Other fruits | 17.2754232 | 4.20739415 | 4.10596741 | 4.7969E-05 |

**Table S2** Model coefficients from robustness check with five similar treatments as specified in Table S1 but with tree cover variables extracted in a 1000m radius circle around each household instead of 2000m.

| Treatment | Covariates | Model | Outcome | Estimate | Std. Error | t value | Pr(>\|t\|) |
| --- | --- | --- | --- | --- | --- | --- | --- |
| tREES1000 | PRIVATE1000+ PLANT1000 + GFR1000 + PFM + Region + Homegarden + Age + Number of crops + TLU + Head of household (man/woman) + Educational level + Household size + Distance to nearest road + MPI | CBPS (lm) | Zinc | 0.0044623 | 0.00092223 | 4.83861474 | 1.85E-06 |
|  |  |  | Vitamin A | 0.00895374 | 0.00103253 | 8.67168784 | 9.84E-17 |
|  |  |  | Iron | 0.0013191 | 0.00055376 | 2.3820827 | 0.01766692 |
|  |  |  | Kcal | 0.00377741 | 0.00078872 | 4.78926607 | 2.34E-06 |
|  |  |  | Protein | 0.00394604 | 0.00113744 | 3.46921947 | 0.00057688 |
|  |  | CBPS (glm, family=quasipoisson) | DDS | 0.0072834 | 0.0012374 | 5.886 | 8.20E-09 |
| PFM1000 | trees1000 + PRIVATE1000+ PLANT1000 + GFR1000 + Region + Homegarden + Age + Number of crops + TLU + Head of household (man/woman) + Educational level + Household size + Distance to nearest road + MPI | CBPS (lm) | Zinc | 0.00905677 | 0.00242855 | 3.72928839 | 0.00021883 |
|  |  |  | Vitamin A | 0.00780382 | 0.00297875 | 2.6198339 | 0.00912142 |
|  |  |  | Iron | 0.00367448 | 0.0014954 | 2.45719224 | 0.01441299 |
|  |  |  | Kcal | 0.00295066 | 0.00222691 | 1.32500118 | 0.18590336 |
|  |  |  | Protein | 0.00312301 | 0.00333411 | 0.93668507 | 0.34946798 |
|  |  | CBPS (glm, family=quasipoisson) | DDS | 0.0143352 | 0.0037895 | 3.783 | 0.000178 |
| GFR1000 | trees1000 + PRIVATE1000+ PLANT1000 + PFM + Region + Homegarden + Age + Number of crops + TLU + Head of household (man/woman) + Educational level + Household size + Distance to nearest road + MPI | CBPS (lm) | Zinc | 0.00020783 | 0.00333715 | 0.06227703 | 0.95037235 |
|  |  |  | Vitamin A | 0.00313136 | 0.00368005 | 0.85090066 | 0.3953176 |
|  |  |  | Iron | -8.16E-05 | 0.00202339 | -0.0403261 | 0.96785265 |
|  |  |  | Kcal | 0.0028804 | 0.0030461 | 0.94560041 | 0.34490532 |
|  |  |  | Protein | -0.001225 | 0.00432793 | -0.2830375 | 0.77728989 |
|  |  | CBPS (glm, family=quasipoisson) | DDS | 0.0023392 | 0.0051687 | 0.453 | 0.6511 |
| PLANT1000 | trees1000 + PRIVATE1000+ GFR1000 + PFM + Region + Homegarden + Age + Number of crops + TLU + Head of household (man/woman) + Educational level + Household size + Distance to nearest road + MPI | CBPS (lm) | Zinc | -0.0075246 | 0.01477089 | -0.509422 | 0.61072854 |
|  |  |  | Vitamin A | 0.01082702 | 0.01671293 | 0.64782309 | 0.51745934 |
|  |  |  | Iron | -0.0040096 | 0.00886129 | -0.4524857 | 0.65115669 |
|  |  |  | Kcal | 0.00282276 | 0.01337113 | 0.21110841 | 0.8329068 |
|  |  |  | Protein | 0.00842333 | 0.01980223 | 0.42537284 | 0.67078646 |
|  |  | CBPS (glm, family=quasipoisson) | DDS | 0.025553 | 0.0275023 | -0.929 | 0.353367 |
| PRIVATE1000 | trees1000 + PLANT1000 + GFR1000 + PFM + Region + Homegarden + Age + Number of crops + TLU + Head of household (man/woman) + Educational level + Household size + Distance to nearest road + MPI | CBPS (lm) | Zinc | -0.0568218 | 0.02983487 | -1.9045427 | 0.05753404 |
|  |  |  | Vitamin A | 0.04691775 | 0.03240411 | 1.4478951 | 0.14840508 |
|  |  |  | Iron | -0.0331408 | 0.0172861 | -1.9171942 | 0.05590285 |
|  |  |  | Kcal | -0.0423324 | 0.02650252 | -1.5972986 | 0.11096396 |
|  |  |  | Protein | -0.053569 | 0.03653911 | -1.4660741 | 0.14338904 |
|  |  | CBPS (glm, family=quasipoisson) | DDS | -1.34E-02 | 4.67E-02 | -0.287 | 0.774314 |
